# Nitric Oxide to Fight Viral Infections

**DOI:** 10.1002/advs.202003895

**Published:** 2021-02-09

**Authors:** Fabio Lisi, Alexander N. Zelikin, Rona Chandrawati

**Affiliations:** ^1^ School of Chemical Engineering and Australian Centre for NanoMedicine (ACN) The University of New South Wales (UNSW Sydney) Sydney NSW 2052 Australia; ^2^ Department of Chemistry and iNANO Interdisciplinary Nanoscience Center Aarhus University Aarhus 8000 Denmark

**Keywords:** coronavirus, COVID‐19, nitric oxide, viral infections, virus

## Abstract

Coronavirus disease 2019 (COVID‐19) is an infectious disease caused by the severe acute respiratory syndrome coronavirus‐2 (SARS‐CoV‐2) that has quickly and deeply affected the world, with over 60 million confirmed cases. There has been a great effort worldwide to contain the virus and to search for an effective treatment for patients who become critically ill with COVID‐19. A promising therapeutic compound currently undergoing clinical trials for COVID‐19 is nitric oxide (NO), which is a free radical that has been previously reported to inhibit the replication of several DNA and RNA viruses, including coronaviruses. Although NO has potent antiviral activity, it has a complex role in the immunological host responses to viral infections, i.e., it can be essential for pathogen control or detrimental for the host, depending on its concentration and the type of virus. In this Essay, the antiviral role of NO against SARS‐CoV, SARS‐CoV‐2, and other human viruses is highlighted, current development of NO‐based therapies used in the clinic is summarized, existing challenges are discussed and possible further developments of NO to fight viral infections are suggested.

## Graphical abstract

Nitric oxide (NO) has a complex role in the immunological host responses to viral infections, i.e., it can be essential for pathogen control or detrimental for the host, depending on its concentration and the type of virus. Inhaled NO therapy has proven to be an efficient treatment against several respiratory viruses and several clinical trials are currently testing its efficiency against COVID‐19.

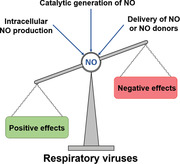

## 1 Introduction

Infections have plagued mankind for centuries and they remain among the leading causes of death and disability worldwide.^[^ ^1^ ^]^ They are caused by pathogenic microorganisms, such as viruses, bacteria, parasites, or fungi. Several factors contribute to the emergence/re‐emergence of infectious diseases, including microbial diversity and evolution, social factors, and environmental conditions.^[^ ^1^, ^2^, ^3^ ^]^ An example of the interaction among these factors is zoonotic diseases, which arise at the interface of human, animal, and environmental health. According to Keusch et al., at least 65% of recent major human infectious disease outbreaks have originated from zoonotic viruses,^[^ ^3^ ^]^ including: acquired immune deficiency syndrome (AIDS), severe acute respiratory syndrome (SARS), Middle East respiratory syndrome (MERS), Ebola, swine influenza, avian influenza, dengue fever, and zika fever. Coronavirus disease 2019 (COVID‐19), a respiratory disease caused by the zoonotic severe acute respiratory syndrome coronavirus‐2 (SARS‐CoV‐2), is the latest example of infectious disease with over 60 million infections and a million deaths globally to date.

**Figure 1:**
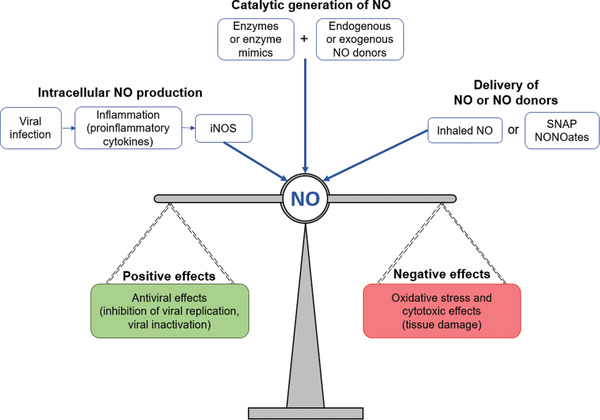
A general overview of the role of nitric oxide (NO) during viral infections, where the radical is involved in the modulation of the immune response. In the body, NO can be: synthesized within various cell types by the enzyme iNOS; generated from endogenous or exogenous NO donors using enzymes or enzyme mimics; delivered by the administration of exogenous NO donors. The overall effect of NO activity can either be positive (pathogen control) or negative (disease promoting), depending on the pathogen, the concentrations of the radical, and for how long NO is produced.

Nitric oxide (NO) is a signaling molecule produced in mammalian cells by NO synthase and it is involved in a wide range of physiological processes, including inflammatory response,^[^ ^4^ ^]^ bronchodilation (relaxation of the smooth muscles in the lungs to open airways),^[^ ^5^, ^6^ ^]^ vasodilation (widening of blood vessels to increase blood flow),^[^ ^7^, ^8^, ^9^ ^]^ modulation of intraocular pressure,^[^ ^10^, ^11^ ^]^ regulation of neuronal function and signal transmission.^[^ ^12^ ^]^ It has been well established that NO has a role in the pathogenesis of many human viral infections^[^ ^13^ ^]^ as well as direct or indirect antiviral activity.^[^ ^14^ ^]^ Direct antiviral activity means that NO can directly inactivate viral particles or inhibit their replication, while indirect activity means a modulation of the host immune response that usually produces an inflammatory response.^[^ ^15^ ^]^ NO can also react with other molecules to produce several reactive oxygen/nitrogen species, including peroxynitrite (ONOO^–^), dinitrogen trioxide (N_2_O_3_), and nitrogen dioxide (NO_2_). These reactive oxygen/nitrogen species themselves may have a direct antiviral effect on the pathogen, but they could also induce oxidative stress that may cause severe cytotoxic effects (**Figure** **1**).^[^ ^15^, ^16^, ^17^ ^]^ Interestingly, whether the overall effect of NO is positive (antiviral) or negative (pathogenic) seems to be virus‐dependent (discussed later in this Essay).

NO activity is also concentration‐dependent. During viral infection, intracellular NO production increases.^[^ ^18^, ^19^ ^]^ While local and controlled production of NO may be beneficial—aiding in the control of infections—generalized activation and overproduction may contribute to tissue damage and cell death—contributing to the pathogenesis.^[^ ^16^, ^20^, ^21^, ^22^, ^23^ ^]^ Therefore, NO production in the host must be tightly controlled to exert antiviral rather than harmful effects.^[^ ^24^, ^25^ ^]^ NO is produced endogenously in a number of cells and tissues by the enzymatic action of NO synthase (NOS), which catalyzes the oxidation of l‐arginine to NO and l‐citrulline.^[^ ^15^, ^26^ ^]^ In mammals, there are three isoforms of NOS: neuronal NOS (nNOS or NOS‐1), inducible NOS (iNOS or NOS‐2), and endothelial NOS (eNOS or NOS‐3).^[^ ^15^, ^26^ ^]^ eNOS and nNOS produce NO only for short periods of time (seconds to minutes) following enzyme activation. In contrast, iNOS produces 10–100 times more NO for comparatively long periods of time (hours to days) and, during an infection, its expression is localized in specific tissues and cells.^[^ ^15^, ^25^ ^]^ It is the iNOS that has been mainly associated with the host response to viral infections.^[^ ^22^, ^27^ ^]^ Because of the high levels of NO that iNOS can produce, regulation of this enzyme is particularly important. Mediators that regulate iNOS expression are proinflammatory cytokines, such as interferon‐*γ* (IFN‐*γ*), interleukin 12 (IL‐12), and tumor necrosis factor‐alpha (TNF‐*α*). The mechanism of this regulation is cell‐dependent and has been demonstrated in several human cells, including hepatocytes, lung epithelial cells, alveolar macrophages, and monocytes.^[^ ^24^, ^28^, ^29^ ^]^ When the iNOS gene is strongly upregulated, the high amount of NO generated might lead to tissue damage. However, defining what concentration of the radical leads to negative effects is not trivial, because of the difficulties involved in the direct quantification of the NO produced in vivo.^[^ ^30^, ^31^ ^]^ NO is highly reactive towards oxyhemoglobin, oxygen, thiols and other radicals, meaning that its half‐life in the body is only 0.1–2 ms in blood and 6 ms–2 s in tissues.^[^ ^30^, ^32^ ^]^ Owing to this short half‐life, the sphere of influence of NO is limited around its origin; using Fick's second law of diffusion, its diffusion distance at half‐life is 5–490 µm.^[^ ^33^ ^]^ Due to this extremely short physiological half‐life in vivo and localized concentration, only techniques such as fluorescence imaging,^[^ ^34^ ^]^ electron paramagnetic resonance (EPR) spectroscopy,^[^ ^35^ ^]^ and electrochemical sensors^[^ ^30^, ^36^ ^]^ have been able to provide some answers. For example, EPR measurements in vivo on the brains of rabies‐infected rats have determined NO concentrations of 1 × 10^−6^ m or less in healthy rats, which increased to ≈12 × 10^−6^ m at day 5 postinfection (onset of the disease) and to ≈30 × 10^−6^ m at day 7.^[^ ^35^ ^]^ However, most studies reported in the literature describe the measurement of NO in humans only indirectly, relying on the quantification of NO metabolites (nitrites and nitrates) in plasma and urine as a surrogate for NO production.^[^ ^16^ ^]^ Another method to infer localized NO production is the detection of iNOS, performed directly on tissue samples via immunohistochemical staining or indirectly via quantification of iNOS mRNA using polymerase chain reaction (PCR).^[^ ^16^, ^25^ ^]^ Measurement of exhaled NO is also performed, as the radical is considered a biomarker of airway inflammation.^[^ ^37^ ^]^ Reported concentrations of NO in the airways of healthy subjects varies between 0.1 and 41 × 10^−9^ m, depending if the gas is measured in the lower or upper respiratory tract, respectively.^[^ ^38^, ^39^, ^40^ ^]^

In this Essay, we highlight the key findings on the production of NO during viral infections and the role of NO in the treatment of SARS‐CoV, SARS‐CoV‐2, and other human viruses. We provide an overview of the current NO‐based therapies used for the treatment of respiratory viral infections and discuss existing challenges.

## 2 NO Production During Viral Infections

A list of experimental studies on the production of NO in response to viral infections is reported in **Table** **1**. Herein, we aim to illustrate the conflicting role of NO (antiviral or pathogenic effects) in viral infections, and the crucial difference between respiratory and non‐respiratory viruses. For a more exhaustive overview of the role of NO on specific diseases, readers are referred to the reviews of Chaturvedi et al.^[^ ^41^ ^]^ (dengue), Akaike et al.^[^ ^42^ ^]^ (influenza), Torre et al.^[^ ^43^ ^]^ (HIV), Proud et al.^[^ ^44^ ^]^ (common cold), and Iwakiri et al.^[^ ^45^ ^]^ (hepatitis).

**Table 1:** In vitro and in vivo studies on the effect of nitric oxide on respiratory and non‐respiratory infections caused by DNA and RNA viruses. ss: single‐stranded, ds: double‐stranded. In preparing this table, priority was given to the selection of in vivo studies in humans

	Family	Viral nucleic acid	Envelope	Virus (common name)	Studies
Respiratory	Coronaviridae	Positive‐sense ssRNA	Yes	Severe acute respiratory syndrome coronavirus (SARS‐CoV)	In vitro^[^ ^17^, ^46^, ^47^ ^]^
				Severe acute respiratory syndrome coronavirus‐2 (SARS‐CoV‐2)	In vitro^[^ ^48^ ^]^
	Picornaviridae	Positive‐sense ssRNA	No	Human rhinovirus	In vitro^[^ ^49^ ^]^ In vivo (humans)^[^ ^27^, ^50^ ^]^
	Orthomyxoviridae	Negative‐sense ssRNA	Yes	Influenza A subtype H1N1 (swine flu)	In vitro^[^ ^51^ ^]^ In vivo (humans)^[^ ^52^ ^]^
				Influenza A subtype H3N2	In vitro^[^ ^24^ ^]^
				Influenza A subtype H2N2	In vivo (mice)^[^ ^53^ ^]^
Non‐respiratory	Flaviviridae	Positive‐sense ssRNA	Yes	Dengue virus	In vitro^[^ ^54^, ^55^ ^]^ In vivo (humans)^[^ ^56^, ^57^, ^58^ ^]^
				Hepatitis C	In vitro^[^ ^59^, ^60^ ^]^ In vivo (humans)^[^ ^61^, ^62^, ^63^ ^]^
	Retroviridae	Positive‐sense ssRNA	Yes	Human immunodeficiency virus (HIV)	In vitro^[^ ^64^, ^65^ ^]^ In vivo (humans)^[^ ^66^, ^67^ ^]^
	Herpesviridae	dsDNA	Yes	Herpes simplex virus	In vitro^[^ ^68^ ^]^ In vivo (mice)^[^ ^20^, ^21^, ^69^, ^70^ ^]^

### 2.1 Respiratory Viruses

Human rhinovirus (HRV) is the predominant cause of the common cold, one of the most frequently experienced acute respiratory illnesses in humans.^[^ ^27^ ^]^ In response to HRV infections, Sanders et al. found elevated iNOS expression in the nasal cells obtained from infected patients.^[^ ^50^ ^]^ This virally induced increase in iNOS expression was associated with higher levels of exhaled NO (≈550% higher compared to the baseline) from both the nasal and lower airways. Interestingly, the increase in exhaled nasal NO levels was inversely related to the symptoms, suggesting that NO may play an important role in the innate immune response to rhinovirus infections, contributing to the resolution of the cold.^[^ ^27^, ^50^ ^]^ Elevated NO levels were also observed in patients infected with influenza A, a respiratory virus responsible for many of the seasonal outbreaks.^[^ ^71^ ^]^ Murphy et al. infected 14 healthy volunteers with influenza A H1N1 and monitored the levels of exhaled NO from the nose and the mouth.^[^ ^52^ ^]^ Only oral NO levels increased, peaking at day 8 postinfection (15.4 ppb). This increase was not associated with patient symptoms, which peaked at day 3 postinfection. This suggested that elevated NO levels may not be directly responsible for the respiratory tract symptoms of infection with influenza A. From this study NO seems not to have harmful effects on the hosts in response to influenza; however, the concentration of exhaled NO measured was much lower compared with HRV infections. Unlike what was observed in humans, in a mouse model infected with influenza A H2N2, Akaike et al. found that NO is crucial for the pathogenesis of pneumonia but not as an antiviral effector mechanism.^[^ ^53^ ^]^ Although studies in mouse models have indicated that the release of NO after infection with influenza viruses contributes to the pathogenesis of pneumonia, it may be argued that the production of NO in the respiratory tract may also provide a first‐line defense mechanism against influenza viruses.^[^ ^24^ ^]^

### 2.2 Non‐respiratory Viruses

In opposition to respiratory viruses, NO production during non‐respiratory infections is mostly associated with pathogenic effects. Hepatitis C is an infectious disease caused by the hepatitis C virus (HCV) that primarily affects the liver. Mihm et al. analyzed liver tissue from chronically HCV‐infected patients, where the iNOS expression was found to be positively correlated to the level of viral RNA.^[^ ^62^ ^]^ A positive correlation was also found between iNOS activity and tissue damage, suggesting a role of NO in the pathogenesis of human chronic viral hepatitis.^[^ ^62^, ^63^ ^]^ Valero et al. first reported the presence of increased NO levels (determined by assaying for nitrite and nitrate) in the serum of humans with dengue fever, a disease caused by the dengue virus.^[^ ^56^ ^]^ Since NO induces blood vessels to relax and dilate, which lowers blood pressure, the authors hypothesized that increased NO in classical dengue could be important in the evolution of the disease into its severe hemorrhagic form.^[^ ^56^ ^]^ NO also has a role in the pathogenesis of pneumonia from herpes simplex virus 1 (HSV‐1). Adler et al. infected mice with HSV‐1 via intranasal inoculation, resulting in the development of pneumonia.^[^ ^21^ ^]^ Some mice were treated with an inhibitor of nitric oxide synthase activity (L‐NMMA), while a control group was not. L‐NMMA treatment significantly improved survival and pulmonary compliance of HSV‐1 infected mice, despite the presence of a 17‐fold higher pulmonary viral titer. Therefore, instead of worsening the disease by diminishing the antiviral effect of NO, iNOS inhibition was beneficial. It reduced the proinflammatory effects of NO, which resulted to be more important for the pathogenesis of HSV‐1 pneumonia than the antiviral effects of NO.^[^ ^20^, ^21^ ^]^

## 3 Antiviral Activity of NO

Antiviral effects of NO on viral infections in vitro and in vivo were first documented in the early 1990s on ectromelia, vaccinia, vesicular stomatitis, and herpes simplex‐1 viruses,^[^ ^68^, ^69^, ^72^, ^73^, ^74^ ^]^ later on for HIV,^[^ ^75^ ^]^ influenza,^[^ ^24^ ^]^ and—most importantly in light of the current pandemic—SARS‐CoV^[^ ^17^, ^46^, ^47^ ^]^ and SARS‐CoV‐2/COVID‐19.^[^ ^48^ ^]^

In itself, this notion is worth highlighting; current understanding in the field is that broad spectrum antivirals are highly warranted but at present are hardly available. From the early days, it was clear that NO exhibits a rather broad spectrum of activity and limits infectivity of diverse viruses, RNA and DNA containing, enveloped and naked. These observations are rather unique among antiviral drugs on the market today.^[^ ^76^ ^]^

Target proteins for the antiviral activity of NO include proteases, reverse transcriptases, ribonucleotide reductases, as well as the zinc‐fingers and related domains.^[^ ^77^ ^]^ Mechanistically, for proteins in which cysteine (Cys) is directly involved in catalysis (e.g., cysteinyl proteases), the mechanism of activity lies in nitrosylation of the amino acid involved in the catalytic process. For other proteins, Cys is found in the overall microenvironment and a seemingly small change via *S*‐nitrosylation exerts a dramatic effect on catalysis. Interestingly, even for the HIV aspartyl protease, NO has a strong inhibitory effect on the protease activity, through nitrosylation of Cys (two per subunit in this homodimeric protein), which appears to be essential for the catalytic activity of the protease.^[^ ^75^ ^]^ Furthermore, HIV reverse transcriptase Cys38 also appears to be important for the overall microenvironment (the finger domain) and nitrosylation decreases the catalytic activity of this enzyme.^[^ ^78^ ^]^

One study we deem worth highlighting is that from Saura et al., in which antiviral activity of NO on Coxsackie virus was deciphered with confidence.^[^ ^79^ ^]^ Coxsackie is a non‐enveloped ssRNA‐virus, which has a cysteinyl protease, indispensable for virus proliferation. The antiviral effect of NO against Coxsackie was shown to proceed via Cys nitrosylation, and changing cysteine for serine (converting the cysteinyl protease into serine protease) rendered the protease NO‐resistant.

Another study that stands out considered that NO can exert antiviral activity on the virion and/or interfere with intracellular viral replication.^[^ ^80^ ^]^ On an example of the hantavirus (Bunyaviridae family, RNA‐virus), this study showed that *S*‐nitroso‐*N*‐acetylpenicillamine (SNAP) and cytokine‐induced NO had strong antiviral effects, whereas incubation of viral stocks in the presence of SNAP had a limited effect on infectivity—thus revealing that NO‐mediated effects are intracellular rather than occurring on the virion. Interestingly, viral titers in iNOS ‐/‐ mice were higher than in control mice, suggesting that NO inhibits virus replication in vivo.

Specifically for SARS‐CoV, to our knowledge, the first report of NO as an inhibitor of viral infectivity was that from Keyaerts et al. in 2004 (using SNAP as a donor, in vitro).^[^ ^47^ ^]^ Later, Åkerström et al. showed that the mechanism of action is at least two‐pronged.^[^ ^17^, ^46^ ^]^ One mechanism is the decreased palmitoylation of the spike (S) protein, a step pivotal for the protein anchoring to the lipid bilayer. The other mechanism is the nitrosylation of viral proteins. Interestingly, nitrosylation documented in this work was detected using the affinity isolation step and capture of nitrotyrosine, and this work confirms tyrosine nitrosylation for the S protein. Nitrosylation of the cysteinyl protease was also experimentally shown, through the analysis of proteolytic degradation of the viral polypeptide, whereby the content of the nucleocapsid N protein was drastically decreased in the presence of SNAP and high molar mass (nonprocessed) polypeptide content was increased. In the case of SARS‐CoV‐2, Akaberi et al. showed that SARS‐CoV‐2 3CL cysteine protease was a possible target of nitrosylation, which led to inhibition of the protease activity and viral replication.^[^ ^48^ ^]^

While all the studies discussed above present antiviral effects of NO generated by donor compounds, one and only study (to our knowledge) presents antiviral effects of gaseous NO. Specifically, Regev‐Shoshani et al. investigated three strains of the influenza virus in vitro and showed that gaseous NO provided highly efficacious antiviral effects.^[^ ^51^ ^]^ NO doses were matched to those approved in the clinic, further highlighting the translational potential of NO gas for antiviral treatment. Remarkably, this study was all but neglected and all studies on antiviral effects of NO that appear in press continue to rely on NO donors.

## 4 NO‐Based Therapies to Fight Viral Infections

Response to non‐respiratory viral infections in humans is often accompanied by an overproduction of endogenous NO, which mostly contributes to pathogenesis. In these cases, the treatment often includes iNOS inhibitor drugs such as L‐NMMA.^[^ ^21^ ^]^ In stark contrast, the production of endogenous NO seems to have a positive (antiviral) effect against human respiratory viruses. It might be hypothesized that for these diseases, when the endogenous levels of NO are not sufficient to achieve antiviral effects, the administration of additional NO may have a positive therapeutic effect.

There are various strategies to administer additional NO to the body; some of these are early stage technologies, while others have made it to the clinics.^[^ ^26^ ^]^ NO can be introduced via catalytic generation using enzymes or enzymes mimics with NO donors endogenously present in the body,^[^ ^26^, ^81^, ^82^ ^]^ as well as through the delivery of exogenous NO‐releasing species^[^ ^83^ ^]^ (Figure 1). Exogenous NO donors have been synthesized and published in great numbers, and several have been translated to the clinic (although, with rare exceptions, not for the treatment of viral diseases).^[^ ^83^, ^84^ ^]^ FDA‐approved exogenous NO donors include nitroprusside for the treatment of congestive heart failure hypertension, isosorbide dinitrate and nitroglycerine for the treatment of acute angina, and latanoprostene bunod to lower intraocular eye pressure in glaucoma patients.^[^ ^85^ ^]^ The vast majority of in vitro studies using NO donors in viral infections employed SNAP, a synthetic *S*‐nitrosothiol that has been shown to inhibit in vitro the replication of influenza A, dengue, herpes, HIV, SARS‐CoV, and SARS‐CoV‐2 viruses.^[^ ^24^, ^46^, ^48^, ^54^, ^65^, ^68^ ^]^ Such a recurring use of SNAP might be justified by its higher stability compared to primary alkyl *S*‐nitrosothiols (RSNOs).^[^ ^83^ ^]^ In most in vitro studies, the donor is added into the cell culture and the biological effects (e.g., inhibition of viral replication) are monitored. However, the authors usually do not quantify the concentration of the NO generated, and therefore the correlation between the therapeutic level of the donor and virus inhibition is not clear. Another important class of synthetic NO donors is diazeniumdiolates (NONOates), for which the rate of decomposition is not influenced by reducing agents or biological tissues.^[^ ^86^ ^]^ 3‐(2‐hydroxy‐2‐nitroso‐1‐propylhydrazino)‐1‐propanamine (PAPA NONOate) has been shown to inhibit both rhinovirus replication and cytokine production in a dose‐dependent fashion.^[^ ^49^ ^]^ The diethylenetriamine‐NO adduct (DETA/NO) has been used in aerosol form for the treatment of acute respiratory distress syndrome in phase I clinical trial.^[^ ^87^ ^]^ However, defining pharmacokinetics of NO in vivo using donor compounds is a highly challenging task, as it requires controlling blood and body residence time of the donor compounds and to factor in the kinetics of NO release in a complex environment such as blood. Furthermore, even the quantification of NO concentration in blood in real time is a stand‐alone challenge. Finally, NO donors are most often administered systemically, which means that an add‐on challenge is found in drug targeting to the diseased tissues.

A facile, powerful alternative is found in direct pulmonary administration of gaseous NO, via inhalation. This is particularly important in the context of the main subject of this discussion, namely the treatment of COVID‐19. Inhalation of gaseous NO has proven successful for the treatment of circulatory and respiratory conditions including pulmonary infections, pulmonary arterial hypertension, recurrent pulmonary infections associated with cystic fibrosis, and tuberculosis.^[^ ^88^, ^89^ ^]^ Traditionally, NO is delivered from gas cylinders connected to a delivery system that monitors in real time the concentration of NO, nitrogen dioxide, and oxygen. Companies that commercialize this technology include Mallinckrodt Pharmaceuticals (INOmax, FDA approved), Praxair Technologies (Noxivent, FDA approved), and Novoteris (Thiolanox). More recently, portable devices that can generate NO on demand—even outside the hospital setting—have been developed.^[^ ^90^ ^]^ Currently, Genosyl by VERO Biotech is the only FDA approved NO‐generating machine, but companies such as Third Pole Therapeutics, Beyond Air, and Bellerophon Therapeutics are also developing similar devices.

The therapeutic range of inhaled NO depends on the disease; for persistent pulmonary hypertension of the newborn, it is recommended a continuous dose of 20 ppm (832 × 10^−9^ m; 1 ppm of NO corresponds to a concentration of 41.59 × 10^−9^ m) for 14 days, with a therapeutic range of 5–80 ppm. Viral infections such as bronchiolitis are usually treated with higher doses of NO (160 ppm, 6654 × 10^−9^ m) for shorter periods (e.g., 30 min, five times a day), while other infections might be treated with even higher concentrations (up to 250 ppm, 10 397 × 10^−9^ m).^[^ ^91^, ^92^ ^]^ Taken together, these data illustrate the most important aspect of inhaled NO, namely that—unlike the systemic use of NO donors—concentration of gaseous NO is literarily dialed to the desired level, which opens up broad opportunities for efficient, personalized treatments. Another highly advantageous aspect associated with the delivery of gaseous NO is that for the treatment of respiratory conditions such as COVID‐19, the drug is delivered directly to the diseased tissue.

We note that the therapeutic effects of inhaled NO, as for every medicinal agent, have to be carefully balanced against the possible side effects. One potential complication of inhaled NO, especially at high doses (above 80 ppm), is methemoglobinemia, which is a condition characterized by an abnormal level of methemoglobin (i.e., oxidized hemoglobin) in the blood. NO oxidizes heme iron (of hemoglobin), changing its iron oxidation state from Fe^2+^ (ferrous) to the Fe^3+^ (ferric) state. The oxidized hemoglobin has a decreased ability to bind oxygen and this leads to an overall reduced ability of the red blood cells to deliver oxygen to the tissues. Other side effects may be due to the products of NO oxidation such as NO_2_ gas. In our Essay, we focus on the effects of NO against diverse viral pathogens and leave the detailed discussion of side effects to specialized reviews on the subject,^[^ ^93^, ^94^ ^]^ as well as to the ongoing clinical trials that evaluate the efficacy of inhaled NO as a treatment for COVID‐19.

Clinical studies conducted during the SARS outbreak reported that inhalation of 30 ppm NO for at least 3 days helped in preventing disease progression.^[^ ^95^ ^]^ In light of these results, NO is currently trialled for patients with COVID‐19, with seventeen active clinical studies as of October 2020.^[^ ^96^ ^]^ The treatment is based on inhalation of gaseous NO in fourteen studies, with some trials focusing on the continuous delivery of low doses of NO, while others using up to 300 ppm of the radical. The remaining three studies are based on the NO donor sodium nitrite, which is administered as a nasal spray (called “nitric oxide releasing solution”, NORS). These strategies may be important to mitigate the symptoms of COVID‐19, especially in view of the current shortage of ventilators.^[^ ^97^ ^]^ The details of each clinical trial are reported in **Table** **2**.

**Table 2:** Active clinical studies (October 2020) that use nitric oxide for the treatment or prevention of COVID‐19. Results collected from the databases ClinicalTrials.gov and ClinicalTrialsRegister.eu. No results were found on ChiCTR.org.cn and ISRCTN registry. ARDS: acute respiratory distress syndrome, iNO: inhaled NO, NORS: NO releasing solution

Identification code	Status	Drug	Administration	Aims	Expected outcomes
EudraCT 2020‐001490‐68	Ongoing	NO	Inhalation	Measure the difference in ventilator treatment time in patients with ARDS.	A decrease in the duration of continuous ventilator treatment of the group treated with iNO compared to the control group.
EudraCT 2020‐002394‐94	Ongoing	NO	Inhalation	Clinical evaluation of iNO for patients with COVID‐19 using clinical and imaging (high resolution computed tomography) endpoints.	Improvement of functional pulmonary imaging parameters, including imaged airway volume, imaged airway resistance, and blood vessel volume.
EudraCT 2020‐001329‐30	Ongoing	NO	Inhalation	Assess the rate of change in oxygenation in mechanically ventilated patients with ARDS.	Increase in oxygenation of the group treated with iNO compared to the control group.
NCT04388683	Recruiting	NO	Inhalation	Investigation of iNO to prevent systemic deoxygenation and inflammation, with escalation to higher levels of oxygen and ventilatory support or death.	Prevention of disease progression (time frame: 28 d). Treatment will be given for up to 14 d unless the patient deteriorates and requires escalation.
NCT04338828	Recruiting	NO	Inhalation	Determine whether iNO improves short term respiratory status and prevents future hospitalization in patients diagnosed with COVID‐19 specifically in the emergency department.	Decreased likelihood to return to the emergency department with worsening symptoms.
NCT04421508	Recruiting	NO	Inhalation	Assess the efficacy of pulsed iNO versus placebo in subjects with mild or moderate COVID‐19 who are hospitalized and require supplemental oxygen without assisted ventilation.	Decreased proportion of subjects who died or had respiratory failure (time frame: day 28).
NCT04397692	Recruiting	NO	Inhalation	Evaluate the efficacy of 80 ppm NO given four times a day in addition to the standard of care of patients.	Increased time to deterioration of respiratory symptoms (time frame: 14 d), determined by escalation to either noninvasive ventilation, high‐flow nasal cannula, or intubation.
NCT04306393	Recruiting	NO	Inhalation	Determine whether iNO improves oxygenation in patients with ARDS.	Improvement of arterial oxygenation at 48 h from enrolment.
NCT04312243	Recruiting	NO	Inhalation	Assess whether intermittent delivery of iNO gas in air at a high dose before and after the work shift may protect healthcare workers from SARS‐CoV‐2 infection.	Decreased percentage of subjects with COVID‐19 diagnosis in the treatment group.
NCT03331445	Recruiting	NO	Inhalation	Assess the efficacy of NO in acting as an antiviral agent, resulting in the reduction of incidence of oxygen therapy.	Reduction in the incidence of mechanical ventilation during the NO treatment.
NCT04383002	Recruiting	NO	Inhalation	Test whether high dose iNO is safe and can reverse virus burden and respiratory failure in patients on mechanical ventilation.	Negative COVID‐19 PCR at the completion of treatment (day 7) from tracheal aspirate.
NCT04456088	Not yet recruiting	NO	Inhalation	Evaluate the efficacy of 150 ppm NO given in addition to the standard of care of patients with COVID‐19.	Decreased time to deterioration (up to 14 d) for patients treated with NO.
NCT04476992	Active, not recruiting	NO	Inhalation	Compare the intermittent versus continuous inhalation of NO in spontaneous breathing patients.	High concentration iNO with an adjunct of continuous low dose administration between the high concentration treatments can be safely administered in hypoxemic COVID‐19 patients compared to the high dose treatment alone.
NCT04305457	Active, not recruiting	NO	Inhalation	Test whether iNO therapy prevents disease progression in spontaneous breathing patients with mild to moderate COVID‐19.	Reduction in the incidence of patients requiring intubation and mechanical ventilation.
NCT04337918	Recruiting	NORS	Gargle + nasal spray + nasopharyngeal flush	Evaluate the efficacy of NORS treatment for the prevention of SARS‐CoV‐2 infections in healthcare workers.	Decreased proportion of subjects with positive COVID‐19 in NORS versus control group by 21 d.
NCT04460183	Recruiting	NORS	Inhalation using a nebulizer	Evaluate the efficacy of NORS in reducing the rate of progression to a more severe level of COVID‐19.	Increased proportion of participants with safe levels of oxygen saturation by day 14 versus standard of care alone.
NCT04443868	Not yet recruiting	NORS	Nasal spray + nasal irrigation	Evaluate the efficacy of NORS to treat and prevent the exacerbation of mild COVID‐19 infections.	Reduction of clinical symptoms as compared to saline placebo.

## 5 Conclusions

Although NO's role in viral infections is still not completely understood, the production of the radical seems to have a positive antiviral effect on respiratory viruses, while mostly leading to pathogenesis in non‐respiratory infections. This difference is further confirmed by the fact that NO‐based therapies (e.g., inhaled NO) have demonstrated success in clinical settings for the treatment of respiratory viruses. Interestingly, the concentration of inhaled NO is in the range of mm, orders of magnitude higher than the endogenous levels (µm–nm). The difference between the concentration of NO produced endogenously by the body and externally administered by NO‐based therapies is an important factor to consider, taking into account that the bioavailability and reactivity of NO dissolved in the tissue might be different from the gaseous one. In light of the above, to develop NO therapies with better outcomes and less side effects, it is important to characterize what is the amount of inhaled NO that locally dissolves in the tissues, and how its final concentration compares to the endogenous levels. Yet, this kind of measurement is particularly challenging in vivo, and more interdisciplinary research in NO‐detection technologies is required.

Taken together, existing evidence of activity of NO against viruses, FDA approval for inhaled NO as prior evidence of safety, and therapeutic efficacy of inhaled NO against pulmonary hypertension and thrombocytopenia strongly suggest that inhaled NO is a unique, readily available agent for the treatment of respiratory infections, including COVID‐19.

## Conflict of Interest

The authors declare no conflict of interest.

## References

[1] D. M. Morens , G. K. Folkers , A. S. Fauci , Nature 2004, 430, 242. 15241422 10.1038/nature02759 PMC7094993

[2] S. Binder , A. M. Levitt , J. J. Sacks , J. M. Hughes , Science 1999, 284, 1311. 10334978 10.1126/science.284.5418.1311

[3] G. T. Keusch , M. Pappaioanou , M. C. Gonzalez , K. A. Scott , P. Tsai , Sustaining Global Surveillance and Response to Emerging Zoonotic Diseases, The National Academies Press, Washington DC 2009. 25009943

[4] F. C. Fang , Nat. Rev. Microbiol. 2004, 2, 820. 15378046 10.1038/nrmicro1004

[5] F. L. M. Ricciardolo , Thorax 2003, 58, 175. 12554905 10.1136/thorax.58.2.175 PMC1746564

[6] P. J. Barnes , Ann. Med. 1995, 27, 389. 7546629 10.3109/07853899509002592

[7] J. O. Lundberg , M. T. Gladwin , E. Weitzberg , Nat. Rev. Drug Discovery 2015, 14, 623. 26265312 10.1038/nrd4623

[8] M. J. Simmonds , J. A. Detterich , P. Connes , Biorheology 2014, 51, 121. 24819865 10.3233/BIR-140653 PMC6441278

[9] A. K. Winther , B. Fejerskov , M. Ter Meer , N. B. S. Jensen , R. Dillion , J. E. Schaffer , R. Chandrawati , M. M. Stevens , L. J. Schultze Kool , U. Simonsen , A. N. Zelikin , ACS Appl. Mater. Interfaces 2018, 10, 10741. 29570264 10.1021/acsami.8b01658 PMC5887086

[10] R. Chandrawati , J. Y. H. Chang , E. Reina‐Torres , C. Jumeaux , J. M. Sherwood , W. D. Stamer , A. N. Zelikin , D. R. Overby , M. M. Stevens , Adv. Mater. 2017, 29, 1604932. 10.1002/adma.201604932 PMC5400071 28221702

[11] J. Y. H. Chang , W. D. Stamer , J. Bertrand , A. T. Read , C. M. Marando , C. R. Ethier , D. R. Overby , Am. J. Physiol. 2015, 309, C205. 10.1152/ajpcell.00347.2014 PMC4537932 26040898

[12] J. Garthwaite , Eur. J. Neurosci. 2008, 27, 2783. 18588525 10.1111/j.1460-9568.2008.06285.x PMC2610389

[13] T. Akaike , Rev. Med. Virol. 2001, 11, 87. 11262528 10.1002/rmv.303 PMC7169086

[14] P. L. Majano , Cell Death Differ. 2003, 10, S13. 12655339 10.1038/sj.cdd.4401115

[15] T. Akaike , H. Maeda , Immunology 2000, 101, 300. 11106932 10.1046/j.1365-2567.2000.00142.x PMC2327086

[16] D. Burgner , K. Rockett , D. Kwiatkowski , Arch. Dis. Child. 1999, 81, 185. 10490536 10.1136/adc.81.2.185 PMC1718000

[17] S. Åkerström , V. Gunalan , C. Tat , Y. Tan , A. Mirazimi , Virology 2009, 395, 1. 19800091 10.1016/j.virol.2009.09.007 PMC7111989

[18] S. Kharitonov , D. Yates , P. Barnes , Eur. Respir. J. 1995, 8, 295. 7538934 10.1183/09031936.95.08020295

[19] L. A. Perrone , J. A. Belser , D. A. Wadford , J. M. Katz , T. M. Tumpey , J. Infect. Dis. 2013, 207, 1576. 23420903 10.1093/infdis/jit062

[20] G. Gamba , H. Cavalieri , M. C. Courreges , E. J. Massouh , F. Benencia , J. Med. Virol. 2004, 73, 313. 15122810 10.1002/jmv.20093

[21] H. Adler , J. L. Beland , N. C. Del‐Pan , L. Kobzik , J. P. Brewer , T. R. Martin , I. J. Rimm , J. Exp. Med. 1997, 185, 1533. 9151890 10.1084/jem.185.9.1533 PMC2196291

[22] C. Bogdan , Nat. Immunol. 2001, 2, 907. 11577346 10.1038/ni1001-907

[23] D. A. Wink , H. B. Hines , R. Y. S. Cheng , C. H. Switzer , W. Flores‐santana , M. P. Vitek , L. A. Ridnour , C. A. Colton , J. Leukocyte Biol. 2011, 89, 873. 21233414 10.1189/jlb.1010550 PMC3100761

[24] G. F. Rimmelzwaan , M. M. Baars , P. de Lijster , R. A. Fouchier , A. D. Osterhaus , J. Virol. 1999, 73, 8880. 10482647 10.1128/jvi.73.10.8880-8883.1999 PMC112914

[25] K. D. Kröncke , K. Fehsel , V. Kolb‐Bachofen , Clin. Exp. Immunol. 1998, 113, 147. 9717962 10.1046/j.1365-2249.1998.00648.x PMC1905037

[26] T. Yang , A. N. Zelikin , R. Chandrawati , Adv. Sci. 2018, 5, 1701043. 10.1002/advs.201701043 PMC6010811 29938181

[27] S. P. Sanders , E. S. Siekierski , S. M. Richards , J. D. Porter , F. Imani , D. Proud , J. Allergy Clin. Immunol. 2001, 107, 235. 11174188 10.1067/mai.2001.112028

[28] K. Asano , C. B. E. Chee , B. Gaston , C. M. Lilly , C. Gerard , J. M. Drazen , J. S. Stamler , Proc. Natl. Acad. Sci. USA 1994, 91, 10089. 7524082 10.1073/pnas.91.21.10089 PMC44963

[29] H. Mühl , C. A. Dinarello , in Nitric Oxide Infect. (Ed.: F. C. Fang ), Kluwer Academic Publishers, New York 2002, pp. 77–94.

[30] C. N. Hall , J. Garthwaite , Nitric Oxide – Biol. Chem. 2009, 21, 92. 10.1016/j.niox.2009.07.002 PMC2779337 19602444

[31] P. N. Coneski , M. H. Schoenfisch , Chem. Soc. Rev. 2012, 41, 3753. 22362308 10.1039/c2cs15271a PMC3341472

[32] J. O. Lundberg , E. Weitzberg , Arterioscler., Thromb., Vasc. Biol. 2005, 25, 915. 15746440 10.1161/01.ATV.0000161048.72004.c2

[33] T. Nagano , T. Yoshimura , Chem. Rev. 2002, 102, 1235. 11942795 10.1021/cr010152s

[34] N. M. Iverson , P. W. Barone , M. Shandell , L. J. Trudel , S. Sen , F. Sen , V. Ivanov , E. Atolia , E. Farias , T. P. McNicholas , N. Reuel , N. M. A. Parry , G. N. Wogan , M. S. Strano , Nat. Nanotechnol. 2013, 8, 873. 24185942 10.1038/nnano.2013.222 PMC4066962

[35] D. C. Hooper , S. T. Ohnishi , R. Kean , Y. Numagami , B. Dietzschold , H. Koprowski , Proc. Natl. Acad. Sci. USA 1995, 92, 5312. 7539914 10.1073/pnas.92.12.5312 PMC41684

[36] R. J. Soto , J. R. Hall , M. D. Brown , J. B. Taylor , M. H. Schoenfisch , Anal. Chem. 2017, 89, 276. 28105839 10.1021/acs.analchem.6b04251 PMC6773264

[37] S. A. Kharitonov , P. J. Barnes , Eur. Respir. J. 2000, 16, 781. 11106225 10.1183/09031936.00.16478100

[38] D. C. Chambers , W. S. Tunniclive , J. G. Ayres , Thorax 1998, 53, 677. 9828855 10.1136/thx.53.8.677 PMC1745302

[39] H. Gerlach , R. Rossaint , D. Pappert , M. Knorr , K. Falke , Lancet 1994, 343, 518. 7906764 10.1016/s0140-6736(94)91465-6

[40] A. J. R. Erit , C. Med , Am. J. Respir. Crit. Care Med. 1996, 153, 1773.8665033

[41] U. C. Chaturvedi , R. Nagar , FEMS Immunol. Med. Microbiol. 2009, 56, 9. 19239490 10.1111/j.1574-695X.2009.00544.x PMC7110348

[42] T. Akaike , H. Maeda , in Nitric Oxide Infect. (Ed: F. C. Fang ), Kluwer Academic Publishers, New York 2002, pp. 397–415.

[43] D. Torre , A. Pugliese , F. Speranza , Lancet Infect. Dis. 2002, 2, 273. 12062993 10.1016/s1473-3099(02)00262-1

[44] D. Proud , Curr. Opin. Allergy Clin. Immunol. 2005, 5, 37. 15643342 10.1097/00130832-200502000-00008

[45] Y. Iwakiri , M. Y. Kim , Trends Pharmacol. Sci. 2015, 36, 524. 26027855 10.1016/j.tips.2015.05.001 PMC4532625

[46] S. Åkerström , M. Mousavi‐Jazi , J. Klingstrom , M. Leijon , A. Lundkvist , A. Mirazimi , J. Virol. 2005, 79, 1966. 15650225 10.1128/JVI.79.3.1966-1969.2005 PMC544093

[47] E. Keyaerts , L. Vijgen , L. Chen , P. Maes , G. Hedenstierna , M. Van Ranst , Int. J. Infect. Dis. 2004, 8, 223. 15234326 10.1016/j.ijid.2004.04.012 PMC7128975

[48] D. Akaberi , J. Krambrich , J. Ling , C. Luni , G. Hedenstierna , J. D. Järhult , J. Lennerstrand , Å. Lundkvist , Redox Biol. 2020, 37, 101734. 33007504 10.1016/j.redox.2020.101734 PMC7505071

[49] S. Sanders , E. Siekierski , J. Porter , S. Richards , Proud D. , J. Virol. 1998, 72, 934. 9444985 10.1128/jvi.72.2.934-942.1998 PMC124563

[50] S. P. Sanders , D. Proud , S. Permutt , E. S. Siekierski , R. Yachechko , M. C. Liu , J. Allergy Clin. Immunol. 2004, 113, 697. 15100676 10.1016/j.jaci.2004.01.755

[51] G. Regev‐Shoshani , S. Vimalanathan , B. McMullin , J. Road , Y. Av‐Gay , C. Miller , Nitric Oxide – Biol. Chem. 2013, 31, 48. 10.1016/j.niox.2013.03.007 PMC7110511 23562771

[52] A. W. Murphy , T. A. E. Platts‐Mills , M. Lobo , F. Hayden , Chest 1998, 114, 452. 9726729 10.1378/chest.114.2.452

[53] T. Akaike , Y. Noguchi , S. Ijiri , K. Setoguchi , M. Suga , Y. M. Zheng , B. Dietzschold , H. Maeda , Proc. Natl. Acad. Sci. USA 1996, 93, 2448. 8637894 10.1073/pnas.93.6.2448 PMC39817

[54] W. Charnsilpa , R. Takhampunya , T. P. Endy , M. P. Mammen , D. H. Libraty , S. Ubol , J. Med. Virol. 2005, 77, 89. 16032750 10.1002/jmv.20418

[55] S. Ubol , T. Chareonsirisuthigul , J. Kasisith , C. Klungthong , Virology 2008, 376, 290. 18455750 10.1016/j.virol.2008.03.030

[56] N. Valero , L. M. Espina , G. Añez , E. Torres , J. A. Mosquera , Am. J. Trop. Med. Hyg. 2002, 66, 762. 12224588 10.4269/ajtmh.2002.66.762

[57] P. C. F. Neves‐Souza , E. L. Azeredo , S. M. O. Zagne , R. Valls‐de‐Souza , S. R. N. I. Reis , D. I. S. Cerqueira , R. M. R. Nogueira , C. F. Kubelka , BMC Infect. Dis. 2005, 5, 64. 16109165 10.1186/1471-2334-5-64 PMC1208887

[58] A. C. Mendes‐Ribeiro , M. B. Moss , M. A. S. Siqueira , T. L. Moraes , J. C. Ellory , G. E. Mann , T. M. C. Brunini , Clin. Exp. Pharmacol. Physiol. 2008, 35, 1143. 18505438 10.1111/j.1440-1681.2008.04970.x

[59] K. Machida , K. T. Cheng , V. M. Sung , K. J. Lee , A. M. Levine , M. M. C. Lai , J. Virol. 2004, 78, 8835. 15280491 10.1128/JVI.78.16.8835-8843.2004 PMC479064

[60] M. Frese , V. Schwärzle , K. Barth , N. Krieger , V. Lohmann , S. Mihm , O. Haller , R. Bartenschlager , Hepatology 2002, 35, 694. 11870386 10.1053/jhep.2002.31770

[61] M. J. Amaro , J. Bartolomé , M. Pardo , T. Cotonat , A. López‐Farré , V. Carreño , J. Med. Virol. 1997, 51, 326. 9093948 10.1002/(sici)1096-9071(199704)51:4<326::aid-jmv11>3.0.co;2-g

[62] S. Mihm , A. Fayyazi , G. Ramadori , Hepatology 1997, 26, 451. 9252158 10.1002/hep.510260228

[63] Ö. Kandemir , A. Polat , A. Kaya , J. Viral Hepat. 2002, 9, 419. 12431203 10.1046/j.1365-2893.2002.00382.x

[64] M. I. Bukrmsky , H. S. L. M. Nottet , H. Schmidtmayerova , L. Dubrovsky , C. R. Flanagan , M. E. Mullins , S. A. Lipton , H. E. Gendelman , J. Exp. Med. 1995, 181, 735. 7530762 10.1084/jem.181.2.735 PMC2191885

[65] J. B. Mannick , J. S. Stamler , E. Teng , N. Simpson , J. Lawrence , J. Jordan , R. W. Finberg , J. Acquired Immune Defic. Syndr. 1999, 22, 1. 10534141 10.1097/00042560-199909010-00001

[66] M. O. Loveless , C. R. Phillips , G. D. Giraud , W. E. Holden , Thorax 1997, 52, 185. 9059483 10.1136/thx.52.2.185 PMC1758484

[67] T. G. Evans , K. Rasmussen , G. Wiebke , J. B. Hibbs, Jr , Clin. Exp. Immunol. 1994, 97, 83. 8033424 10.1111/j.1365-2249.1994.tb06583.x PMC1534784

[68] K. D. Croen , J. Clin. Invest. 1993, 91, 2446. 8390481 10.1172/JCI116479 PMC443304

[69] G. Karupiah , Q.‐W. Xie , R. Mark , L. Buller , C. Nathan , C. Duarte , J. D. Macmicking , Science 1993, 261, 1445. 7690156 10.1126/science.7690156

[70] U. Meyding‐Lamadé , J. Haas , W. Lamadé , K. Stingele , R. Kehm , A. Fäth , K. Heinrich , B. Storch Hagenlocher , B. Wildemann , Neurosci. Lett. 1998, 244, 9. 9578132 10.1016/s0304-3940(98)00115-3

[71] J. McGill , J. W. Heusel , K. L. Legge , J. Leukocyte Biol. 2009, 86, 803. 19643736 10.1189/jlb.0509368 PMC2752015

[72] Z. Bi , C. S. Reiss , J. Virol. 1995, 69, 2208. 7533852 10.1128/jvi.69.4.2208-2213.1995 PMC188889

[73] N. Harris , R. M. Buller , G. Karupiah , J. Virol. 1995, 69, 910. 7529336 10.1128/jvi.69.2.910-915.1995 PMC188659

[74] G. Karupiah , N. Harris , J. Exp. Med. 1995, 181, 2171. 7539042 10.1084/jem.181.6.2171 PMC2192048

[75] T. Persichini , M. Colasanti , G. M. Lauro , P. Ascenzi , Biochem. Biophys. Res. Commun. 1998, 250, 575. 9784385 10.1006/bbrc.1998.9350

[76] E. De Clercq , G. Li , Clin. Microbiol. Rev. 2016, 29, 695. 27281742 10.1128/CMR.00102-15 PMC4978613

[77] M. Colasanti , T. Persichini , G. Venturini , P. Ascenzi , IUBMB Life 1999, 48, 25. 10791912 10.1080/713803459

[78] T. Persichini , M. Colasanti , M. Fraziano , V. Colizzi , C. Medana , F. Polticelli , G. Venturini , P. Ascenzi , Biochem. Biophys. Res. Commun. 1999, 258, 624. 10329434 10.1006/bbrc.1999.0581

[79] M. Saura , C. Zaragoza , A. McMillan , R. A. Quick , C. Hohenadl , J. M. Lowenstein , C. J. Lowenstein , Immunity 1999, 10, 21. 10023767 10.1016/S1074-7613(00)80003-5 PMC7129050

[80] J. Klingström , S. Åkerström , J. Hardestam , M. Stoltz , M. Simon , K. I. Falk , A. Mirazimi , M. Rottenberg , Å. Lundkvist , Eur. J. Immunol. 2006, 36, 2649. 16955520 10.1002/eji.200535587 PMC7163486

[81] T. Yang , A. S. Fruergaard , A. K. Winther , A. N. Zelikin , R. Chandrawati , Small 2020, 16, 1906744. 10.1002/smll.201906744 32141238

[82] T. Yang , A. N. Zelikin , R. Chandrawati , Small 2020, 16, 1907635. 10.1002/smll.201907635 32372556

[83] P. G. Wang , M. Xian , X. Tang , X. Wu , Z. Wen , T. Cai , A. J. Janczuk , Chem. Rev. 2002, 102, 1091. 11942788 10.1021/cr000040l

[84] A. W. Carpenter , M. H. Schoenfisch , Chem. Soc. Rev. 2012, 41, 3742. 22362384 10.1039/c2cs15273h PMC3341526

[85] DrugBank , https://www.drugbank.ca/categories/DBCAT000756 (accessed: 2020).

[86] L. K. Keefer , Annu. Rev. Pharmacol. Toxicol. 2003, 43, 585. 12415121 10.1146/annurev.pharmtox.43.100901.135831

[87] C. F. Lam , P. V. Van Heerden , J. Blott , B. Roberts , K. F. Ilett , J. Crit. Care 2004, 19, 48. 15101006 10.1016/j.jcrc.2004.02.009

[88] A. Vazquez‐Torres , F. C. Fang , in Nitric Oxide Infect. (Ed: F. C. Fang ), Kluwer Academic Publishers, New York 2002, pp. 475–488.

[89] P. Bhatraju , J. Crawford , M. Hall , J. D. Lang , Nitric Oxide – Biol. Chem. 2015, 50, 114. 10.1016/j.niox.2015.08.007 26335378

[90] B. Yu , S. Muenster , A. H. Blaesi , D. B. Bloch , W. M. Zapol , Sci. Transl. Med. 2015, 7, 294ra107. 10.1126/scitranslmed.aaa3097 26136478

[91] Beyond Air Inc. , https://www.beyondair.net/technology (accessed: 2020).

[92] U.S. Food and Drug Administration , https://www.accessdata.fda.gov/scripts/cder/daf/index.cfm?event=BasicSearch.process (accessed: 2020).

[93] B. Yu , F. Ichinose , D. B. Bloch , W. M. Zapol , Br. J. Pharmacol. 2019, 176, 246. 30288739 10.1111/bph.14512 PMC6295404

[94] M. Barnes , E. J. Brisbois , F. Radic , Biol. Med. 2020, 152, 422. 10.1016/j.freeradbiomed.2019.11.029 31785330

[95] L. Chen , P. Liu , H. Gao , B. Sun , D. Chao , F. Wang , Y. Zhu , G. Hedenstierna , C. G. Wang , Clin. Infect. Dis. 2004, 39, 1531. 15546092 10.1086/425357 PMC7107896

[96] ClinicalTrials.gov , https://clinicaltrials.gov/ct2/results?cond=Covid19&term=Nitric+Oxide&cntry=&state=&city=&dist= (accessed: 2020).

[97] J. Martel , Y. F. Ko , J. D. Young , D. M. Ojcius , Microbes Infect. 2020, 22, 168. 32387333 10.1016/j.micinf.2020.05.002 PMC7200356

